# Mechanical Strength and Biocompatibility of Ultrafine-Grained Commercial Purity Titanium

**DOI:** 10.1155/2013/914764

**Published:** 2013-07-02

**Authors:** Yuri Estrin, Hyoun-Ee Kim, Rimma Lapovok, Hoi Pang Ng, Ji-Hoon Jo

**Affiliations:** ^1^Centre for Advanced Hybrid Materials, Department of Materials Engineering, Monash University, Clayton, VIC 3800, Australia; ^2^WCU Hybrid Materials Program, Department of Materials Science and Engineering, Seoul National University, Seoul 151-744, Republic of Korea

## Abstract

The effect of grain refinement of commercial purity titanium by equal channel angular pressing (ECAP) on its mechanical performance and bone tissue regeneration is reported. *In vivo* studies conducted on New Zealand white rabbits did not show an enhancement of biocompatibility of ECAP-modified titanium found earlier by *in vitro* testing. However, the observed combination of outstanding mechanical properties achieved by ECAP without a loss of biocompatibility suggests that this is a very promising processing route to bioimplant manufacturing. The study thus supports the expectation that commercial purity titanium modified by ECAP can be seen as an excellent candidate material for bone implants suitable for replacing conventional titanium alloy implants.

## 1. Introduction

Titanium alloys take a prominent place among the materials used for medical implants, particularly in bone replacement and dental implantology [1–5]. However, concerns about possible biotoxicity of alloying elements, such as Al and V in the popular Ti-6Al-4V alloy, have been recently driving the development of *commercial purity* (CP) grades of titanium as an alternative to alloys [6]. This is of particular significance for dental applications, which pose stringent requirements on biocompatibility of the implant materials [5].

If commercial purity (CP) titanium is to replace Ti alloys as a competitive implant material, the loss of strength due to lack of alloying elements needs to be compensated for. The only tool the metallurgist is left with is to modify the microstructure, and mechanical working leading to grain refinement appears to be the only viable option. The severe plastic deformation technique of equal channel angular pressing (ECAP) was proposed as a potent way to achieve strengthening of CP Ti by extreme grain refinement down to deep submicron range [1, 7–9]. It was further shown that such grain refinement also leads to enhanced attachment of living cells to the surface of Ti. Indeed, *in vitro* tests demonstrated that proliferation of the fibroblast [7, 8], preosteoblast [9, 10], and stem cells [11] on the surface of ECAP-modified Ti was markedly increased. The degree of preosteoblast attachment and rate of growth on the surface of CP titanium was also found to be increased as a result of grain refinement by high-pressure torsion—another severe plastic deformation technique [12]. The mechanisms of this effect found *in vitro* are not yet understood, but the first data suggest that it is a change in Ti surface morphology [11] and grain orientation [12] that gives rise to the enhanced cell response. 

As improved biocompatibility *in vitro* is no guarantee that this favourable effect will be replicated in an *in vivo* situation, we have conducted an *in vivo* study on the same Grade 2 CP titanium that was investigated earlier [9, 11]. In this communication, we report the results of a study of the extreme grain refinement by ECAP on the *in vivo* performance of the material in an animal test. For completeness, transmission electron microscopy and mechanical characteristics of the material are presented. 

## 2. Materials and Methods

**Figure 1:**
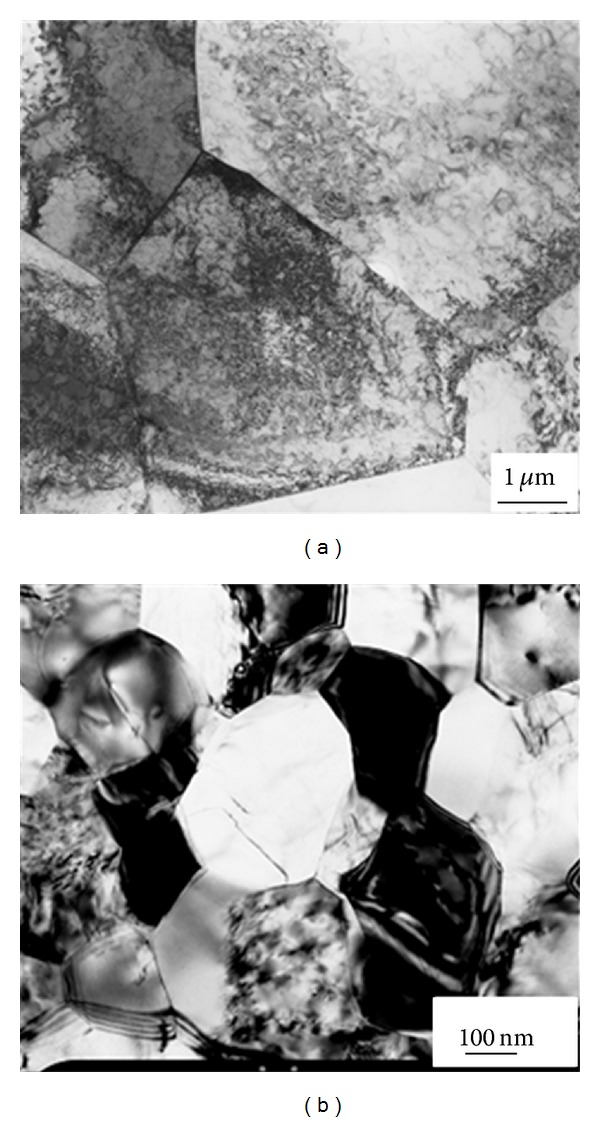
Grain structure of Grade 2 CP Ti in the as-received (a) and the ECAP-modified (b) state.

ASTM Grade 2 CP titanium (Tico Inc., USA), which was also employed in previous work [9, 11], was investigated. Specimens of the material in the as-received condition (extruded bar annealed at 704.4°C for 1 h) as well as in the ECAP-modified state (four ECAP passes at 350°C, route *B*_*c*_ involving rotation by 90° about the long axis between the passes) were used. A back pressure applied made it possible to conduct ECAP at a relatively low temperature of 350°C thus leading to a very substantial level of grain refinement. A huge difference in the average grain size between these two conditions (4.5 *μ*m for the as-received and ca. 200 nm for the ECAP-modified state) is seen from the micrographs in Figure 1.

**Figure 2:**
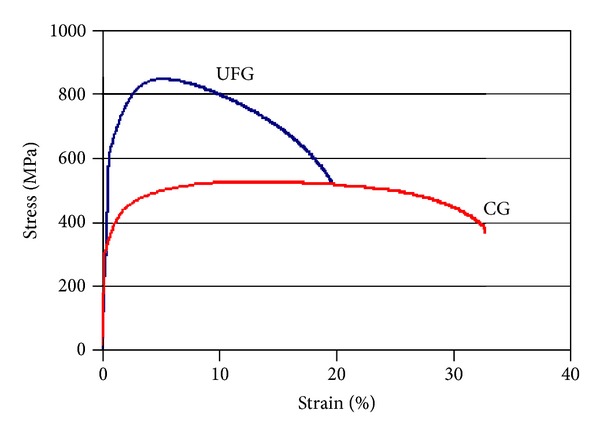
Uniaxial stress-strain curves for the as-received coarse-grained (CG) and ECAP-modified ultrafine-grained (UFG) Grade 2 CP Ti.

This difference in microstructure is responsible for a very strong difference in the tensile deformation behaviour presented in Figure 2. The magnitude of the ultimate tensile strength (about 850 MPa) is in excess of what has ever been achieved for Grade 2 CP Ti by ECAP processing only, that is, without any postprocessing steps [13]. 

The mechanisms of extreme grain refinement by severe plastic deformation by formation of dislocation cell arrangement, which gradually transforms into a new, finer, grain structure upon continued straining, are understood reasonably well [14], as is an inverse correlation between the tensile strength and the grain size. Using a rather robust relation applicable to ultrafine-grained titanium and its alloys [15], the expected fatigue strength (i.e., the stress amplitude corresponding to 10^7^ fatigue cycles) can be estimated roughly as half the ultimate tensile strength, which yields a value of about 425 MPa. While tensile ductility had to be partly sacrificed to gain these levels of strength (which are comparable with those for conventional, coarse-grained Ti-6Al-4V alloy), the remaining value of elongation to failure of about 20% is sufficiently high for biomedical applications in sight. 

As reported earlier [9], the ECAP processing and the concomitant changes in the grain size of the bulk and the morphology of the polished surface of this Grade 2 CP titanium yielded a spectacular (nearly 20-fold!) gain in the proliferation rate of preosteoblast cells as observed *in vitro*. A recent study of the response of human bone marrow-derived mesenchymal stem cells (hMSCs) to the same material showed that the attachment and spreading of hMSCs within the first 24 h of *in vitro* culturing was markedly accelerated on the surface of the ECAP-modified material [11]. Along with the observation of accelerated proliferation of bacteria on ECAP-modified CP Ti [16], these findings indicate clearly that ECAP processing leads to enhanced bioactivity of titanium. These findings are in line with the general trend of nanostructuring being a promoter of improved cell adhesion to and proliferation on titanium [3, 12, 17]. 

With this promise in mind, we have conducted an animal test aiming at an assessment of the extreme grain refinement by ECAP on the *in vivo *biocompatibility of Grade 2 CP titanium. Indeed, it is not uncommon that the *in vivo* behaviour of a candidate implant material is different from the *in vitro* response [18]. In another context, Toledo and Wahl made this point through a pun in the title of their review paper: “*in vitro* hypothesis, *in vivo veritas*” [19].

**Figure 3:**
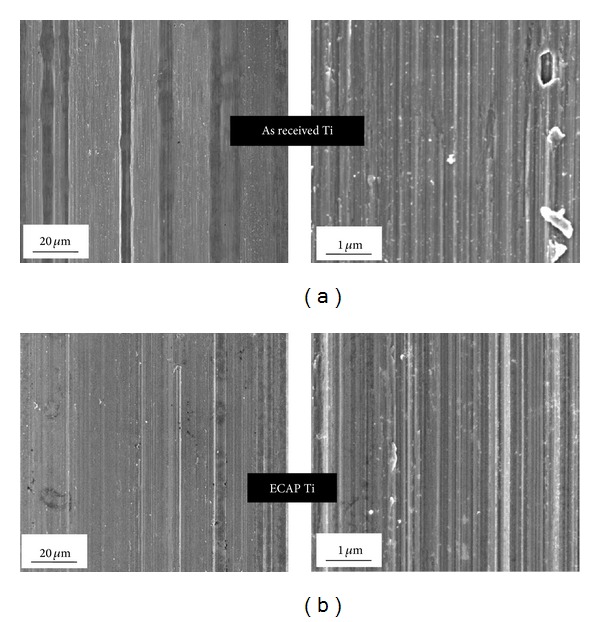
Low-voltage SEM micrographs of the space between the ridges on the threaded parts of as-received (a) and ECAP (b) titanium screws.

**Figure 4:**
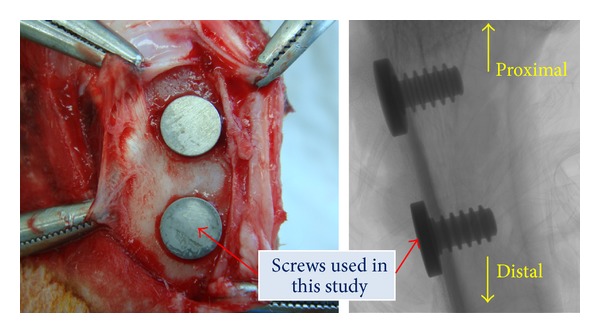
Titanium screws implanted in the rabbit tibia. (Right: CT scan.)

Four New Zealand white rabbits were used as test objects. Screws made from the as-received and the ECAP-modified titanium were CNC machined and implanted in the tibia of the hind legs of a rabbit after ultrasonic cleaning in a distilled water bath for 1 h and sterilisation by *γ*-radiation.  Figure 3 shows the surface morphologies of the screws imaged by a JEM-7001F FEG scanning electron microscope (SEM) operated at 5 kV. The machined surfaces of the as-received and ECAP-modified screws were characterised by a dense array of circumferential grooves, each having a width in the range of 80–110 nm. High resolution edge-on images of the screw surface profile further revealed that these nanoscale grooves typically had a peak-to-peak distance of the order of tens of nanometres. No significant difference could be identified between the two types of screws in terms of the extent of roughness in the micron-to-submicron range, that is, the microroughness. The animals were anesthetised with a combination of 1.5 cc of 2% xylazine HCl (Rompun, Bayer Korea) and 0.5 cc of tiletamine HCl (Zoletil, Virbac Laboratories, France) and lidocaine (Yuhan Corporation, Republic of Korea). Additionally, 1 : 100,000 epinephrine was injected for the local anesthesia. Two defects were made on each tibia using a 3 mm diameter trephine drill. The screw samples were implanted into the tibial bone defects. After the surgery, the wounds were sutured with Surgisorb (Samyang Ltd., Republic of Korea), and then cephradine (Bayer Korea), an antibiotic, was injected into the rabbit for 3 days. Only the screws implanted in the lower (distal) portion of the tibia, where the cortical bone thickness was sufficiently uniform, were used in the present test. (The screws implanted in the upper (proximal) parts of the tibia were not suitable, as the cortical bone thickness is not sufficiently uniform there. These were used for separate tests not reported here.) Hence, a total of four screws of each kind were investigated (Figure 4). While this cannot be considered as a statistically large sample, the animal ethics considerations had priority, and only four animals were sacrificed for the test. They were euthanised four weeks after surgery. The test was carried out in compliance with the strict animal ethics regulations of the Seoul National University. 

## 3. Results and Discussion

**Figure 5:**
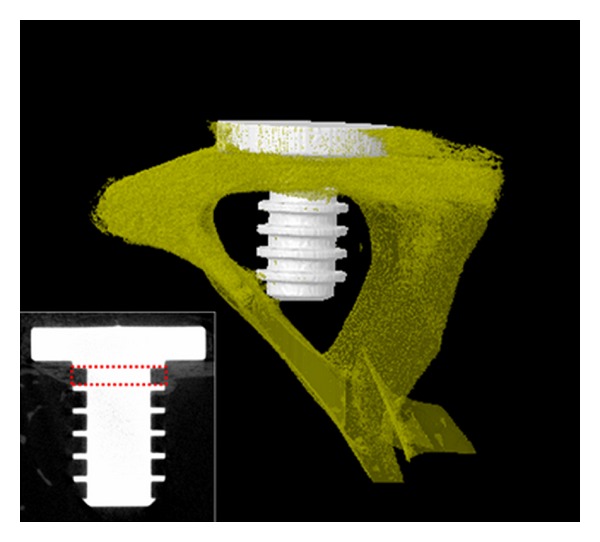
3D micro-CT scan of bone tissue grown on a screw from ECAP-modified titanium. The sketch in the insert defines the volume of interest (VOI) delineated by the red dashed line.

**Figure 6:**
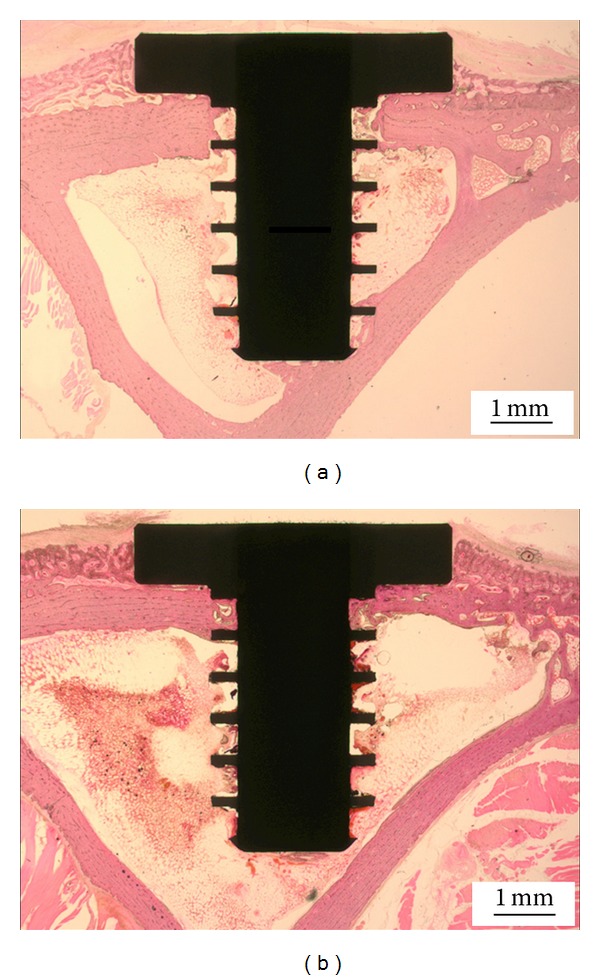
2D histology images of bone tissue growth on the implanted titanium screws. (a) As-received titanium; (b) ECAP-modified titanium.

After four weeks of implantation, bone growth was inspected by histological and microtomography study. The samples were scanned using a micro-CT (Skyscan 1173 X-ray Microtomography System, Skyscan, Belgium) at a resolution of 12 *μ*m, a voltage of 130 kV, and a current of 40 *μ*A. The obtained scans were used for 3D reconstruction by means of a commercial program. The implant and the bone tissue were observed using Data Viewer (Skyscan, Belgium). The results of a quantitative assessment of bone tissue growth on the screws made from the as-received and the ECAP-modified titanium are presented in two ways. (i) The regenerated *bone volume* calculated using micro-CT scans (Skyscan 1173 X-ray Microtomography System, Skyscan, Belgium) is shown in Figure 5. This quantity is defined as the ratio of the bone tissue volume within the volume of interest (VoI, specified in the insert in Figure 5) to the difference between the VoI and the volume of the implant material therein. It is expressed in percent. (ii) In addition, the bone-implant contact (BIC), also expressed in percent, was calculated using histological images. After micro-CT scans, the extracted bone samples were fixed in a neutral 10% formaldehyde solution, and the tissue blocks were embedded in a resin for histological analysis. The blocks were cut into sections and the microscopic images of the trichrome and haematoxylin-eosin stained sections were obtained using Axioskop microscopy (Olympus BX51, Olympus Corporation, Tokyo, Japan). Representative 2D histological sections are shown in Figure 6. 

From these histological images, the BIC ratios were calculated, along with the bone volume, using a digital image analysis program (SPOT, Diagnostic Instruments Inc., MI, USA). Hence, the bone volume was obtained both by micro-CT and by histological investigations, while BIC was derived only by histology—arguably the more appropriate technique for determining this measure of bone regeneration. The appearance of the spreading of the bone tissue into the root space of the screw thread (Figure 6) suggests that cell bone attachment and proliferation are qualitatively similar for both screw materials. This is supported by quantitative data; see below.

**Figure 7:**
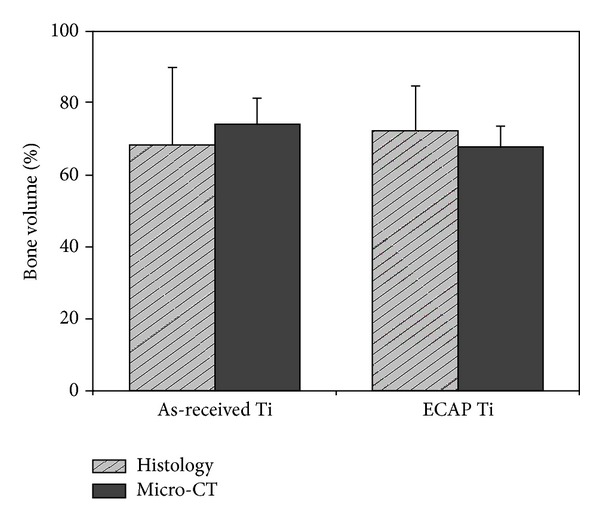
Bone volume (as defined in the text) for the as-received and ECAP-modified titanium screws.

**Figure 8:**
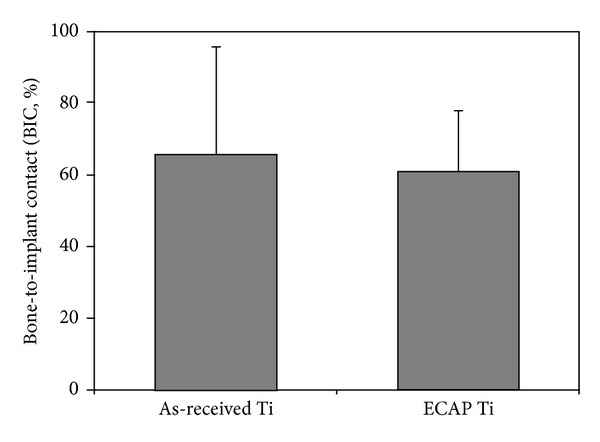
Bone-implant contact (BIC) for the as-received and the ECAP-modified titanium screws.

The diagrams showing the results of micro-CT and histological analysis in terms of these two quantitative measures of bone growth are presented in Figures 7 and 8. Due to the large scatter in the data and a small sample of only four implants in each condition investigated, one cannot establish with certainty which of the two conditions leads to a higher level of bone growth. In terms of their response to cell attachment and proliferation, both types of screws were largely the same. The absence of any major difference in biocompatibility between the two types of screws can be attributed to their striking similarity with respect to the microroughness of the machined surface—despite a very large difference between the as-received and the ECAP-modified screws in grain size in the bulk (4.5 *μ*m versus 200 nm). 

In earlier research, nano- or submicron scale surface topography features were shown to play a crucial role in the adhesion and proliferation of bone cells on titanium [20]. The well-polished surfaces in our previous *in vitro *assays [9, 11, 16] tended to inherit some features of the ultrafine crystallinity of the bulk produced by severe plastic deformation of the material. This kind of surface topography was shown [20] to have a favourable effect on the attachment and proliferation of osteoblast cells—although the exact mechanism for the effect is yet to be uncovered. However, as suggested by SEM results (Figure 3), this difference in surface topography was apparently lost in the process of machining of the implants, so that no significant difference in the *in vivo* cell response to the implants made from coarse-grained and ultrafine-grained Ti was observed.

Assuming no significant differences in surface chemistry of the initial and the ECAP-modified material are introduced by machining, the similarity in surface topography of the machined implants can be seen as the reason for cellular response to the implants not being distinctly different *in vivo*. Nonetheless, even though the present *in vivo* tests did not show enhanced cell response to ECAP-modified implants found earlier by *in vitro* assays [9], it is fair to say that the screws made from ECAP-modified Ti are on par with their counterparts made from the as-received material. 

## 4. Conclusions

The results of the *in vivo* tests show that ECAP processing does not live to the expectations of significant enhancement of bone tissue growth raised by the *in vitro* studies [9]. However, one should not be discouraged by the outcomes of the animal tests. Indeed, the present study has shown that the strength level of about 850 MPa, which is exceptionally high for Grade 2 titanium, can be achieved by ECAP processing without a loss in osseointegration capability of the implants. Thus, this study has demonstrated that the improvement of the mechanical performance of CP titanium by equal channel angular pressing does not compromise its biocompatibility in a real *in vivo* situation. Considering the notion that the surface topography of an implant has a crucial effect on cell attachment, possible correlation between bulk microstructure and surface topography of titanium is of practical interest and thus warrants further investigation. Machined implants commonly undergo further surface treatment before actual implantation [21]. The effect of the various surface treatment processes, to which the initial and the ECAP-modified titanium may or may not respond differently, is thus an important subject for further study—both *in vitro *and *in vivo*.
